# Heatmap3: an improved heatmap package with more powerful and convenient features

**DOI:** 10.1186/1471-2105-15-S10-P16

**Published:** 2014-09-29

**Authors:** Shilin Zhao, Yan Guo, Quanhu Sheng, Yu Shyr

**Affiliations:** 1 Center for Quantitative Sciences, Vanderbilt University, Nashville, TN 37027, USA

## Background

Heat map and clustering are used frequently in expression analysis studies for data visualization. Simple clustering and heat map can be produced from the “heatmap” function in R language. However, it has some limitations in producing advanced graphics and is not highly customizable. Thus, we developed an R package heatmap3 which significantly improves the original heatmap by adding more powerful and convenient features and providing a highly customizable interface.

## Materials and methods

The heatmap3 package is developed based on the heatmap function in the R language and is completely compatible with it. All the commands and parameters for heatmap can also be used in heatmap3. At the same time, heatmap3 imported many new parameters to provide more powerful features. Heatmap3 also provided a highly customizable interface for users’ own functions so that they can label and annotate the data in the figure very easily.

## Results

**Figure 1:**
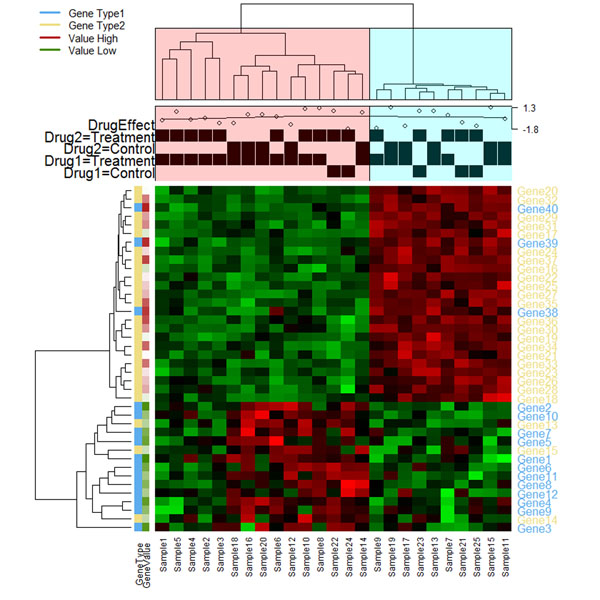
An example of heatmap3 package. The tree in column 1 was divided into two parts based on the correlation between samples and then labeled respectively. The figure in the column side demonstrates the annotation and labeling function for continuous and factor variables. The figure to the left of the rows is an example for multiple layers of phenotypes labeling by color bar.

The new features of heatmap3 include a highly customizable legend and side annotation, a wider range of color selections, new labeling features which allow the user to define multiple layers of phenotypes and automatically compute associations based on these phenotypes. Additional features such as different agglomeration methods for estimating distance between two samples are added for clustering. A simulated data is used to demonstrate the result of heatmap3 package (Figure 1).

## Availability

The heatmap3 package can be accessed freely at github [1].
